# Supplementary material for: Virus shedding kinetics and unconventional virulence tradeoffs
Source: PLoS Pathog. 2021 May 10;17(5):e1009528. doi: 10.1371/journal.ppat.1009528 (PMC8109835; doi:10.1371/journal.ppat.1009528)
Supplement: S1 Text — Figure A shows individual fish viral shedding profiles and Tables A-L shows selected minimal models from statistical analyses. (DOCX) [file ppat.1009528.s001.docx]

**Supporting Information**

***I. Figures***

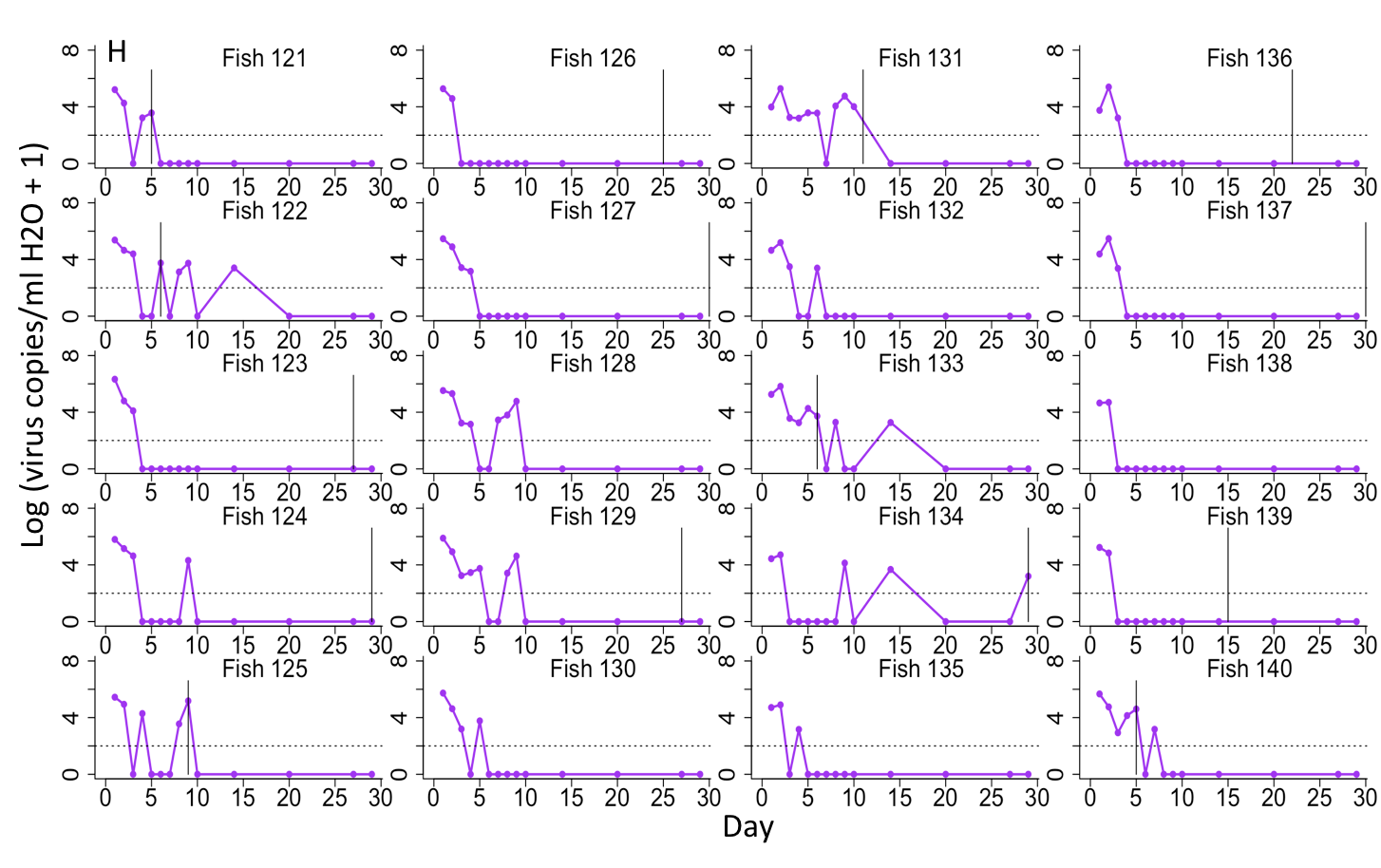


Figure A. Shedding kinetics of individual fish. Each panel shows the quantity of virus shed (viral RNA copies/ml H_2_0) of individual fish, through time, for IHNV genotypes HV (A, C, G; red line), LV (B, C; blue line), LR80 (D, F, H; purple line), and MER95 (E, F; green line), in experiments 1 (A-C), 2 (D-F), and 3 (G-H). Panels with solid lines (A, B, D, E, G, H) denote fish exposed to a single IHNV genotype and panels with dotted lines (C, F) denote fish simultaneously exposed to two genotypes. The fish number is shown at the top of each graph, indicating that for mixed infections, the viral quantities for both genotypes came from the same fish. Mixed infections were not assessed in experiment 3. Points represent the days samples were taken and processed. Vertical lines indicate the day a fish was found dead. Viral RNA detected after the time of death was excluded from all analyses. The horizontal dotted line indicates the detection threshold of the qPCR method.

**II. *Statistical Results***

**Table A: Statistical results number of fish shedding analysis experiment 1.**

Generalized linear mixed model fit by maximum likelihood (Laplace Approximation) ['glmerMod']

Family: binomial ( logit )

Formula: inf.status ~ gen + day + gen:day + (day | fish)

AIC BIC logLik deviance df.resid

793.9 827.7 -389.9 779.9 927

Scaled residuals:

Min 1Q Median 3Q Max

-2.18 -0.48 -0.03 0.51 18.41

Random effects:

Groups Name Variance Std.Dev. Corr

fish (Intercept) 0.62 0.78

day 0.04 0.20 -0.83

Number of obs: 934, groups: fish, 60

Fixed effects:

Estimate Std. Error z value Pr(>|z|)

(Intercept) 2.15 0.35 6.18 6.59e-10 ***

genLV -0.15 0.41 -0.36 0.72

day -0.27 0.07 -3.92 8.97e-05 ***

genLV:day -0.31 0.08 -3.93 8.31e-05 ***

---

Signif. codes: 0 ‘***’ 0.001 ‘**’ 0.01 ‘*’ 0.05 ‘.’ 0.1 ‘ ’ 1

Correlation of Fixed Effects:

(Intr) genLV day

genLV -0.66

day -0.88 0.53

genLV:day 0.51 -0.85 -0.54

**Table B: Statistical results number of fish shedding analysis experiment 2.**

Generalized linear mixed model fit by maximum likelihood (Laplace Approximation) ['glmerMod']

Family: binomial ( logit )

Formula: inf.status ~ gen + day + gen:day + (day | fish)

AIC BIC logLik deviance df.resid

1101.3 1137.0 -543.6 1087.3 1214

Scaled residuals:

Min 1Q Median 3Q Max

-2.71 -0.47 -0.01 0.50 12.81

Random effects:

Groups Name Variance Std.Dev. Corr

fish (Intercept) 0.10 0.31

day 0.01 0.10 -1.00

Number of obs: 1221, groups: fish, 60

Fixed effects:

Estimate Std. Error z value Pr(>|z|)

(Intercept) 2.35 0.22 10.56 < 2e-16 ***

genMER95 -0.56 0.32 -1.77 0.08 .

day -0.14 0.03 -4.92 8.55e-07 ***

genMER95:day -0.24 0.05 -5.38 7.27e-08 ***

---

Signif. codes: 0 ‘***’ 0.001 ‘**’ 0.01 ‘*’ 0.05 ‘.’ 0.1 ‘ ’ 1

Correlation of Fixed Effects:

(Intr) genMER95 day

genMER95 -0.69

day -0.78 0.52

genMER95:dy 0.45 -0.82 -0.54

convergence code: 0

Model failed to converge with max|grad| = 0.014 (tol = 0.001, component 1)

(determined not to be fatal flaw).

**Table C: Statistical results number of fish shedding analysis experiment 3.**

Generalized linear mixed model fit by maximum likelihood (Laplace Approximation) ['glmerMod']

Family: binomial ( logit )

Formula: inf.status ~ day + (day | fish)

AIC BIC logLik deviance df.resid

381.8 402.2 -185.9 371.8 428

Scaled residuals:

Min 1Q Median 3Q Max

-1.78 -0.40 -0.022 0.47 4.43

Random effects:

Groups Name Variance Std.Dev. Corr

fish (Intercept) 2.23 1.49

day 0.29 0.54 -0.97

Number of obs: 433, groups: fish, 40

Fixed effects:

Estimate Std. Error z value Pr(>|z|)

(Intercept) 3.05 0.53 5.81 6.13e-09 ***

day -0.76 0.15 -5.18 2.23e-07 ***

---

Signif. codes: 0 ‘***’ 0.001 ‘**’ 0.01 ‘*’ 0.05 ‘.’ 0.1 ‘ ’ 1

Correlation of Fixed Effects:

(Intr)

day -0.93

**Table D: Statistical results shedding intensity analysis experiment 1.**

Linear mixed-effects model fit by REML

AIC BIC logLik

748.74 795.44 -362.37

Random effects:

Formula: ~day | fish

Structure: General positive-definite, Log-Cholesky parametrization

StdDev Corr

(Intercept) 0.11 (Intr)

day 0.04 -1

Residual 0.01

Correlation Structure: AR(1)

Formula: ~1 | fish

Phi parameter estimate: 0.45

Variance function:

Structure: Power of variance covariate

Formula: ~fitted(.)

power parameter estimate: 3.06

Fixed effects: log.vl ~ gen + comp + day + gen:comp + gen:day

Value Std.Error DF t-value p-value

(Intercept) 4.34 0.13 304 33.55 0.00

genLV -0.003 0.18 304 -0.03 0.99

compMix 0.06 0.13 58 0.47 0.64

day -0.09 0.02 304 -5.53 0.00

genLV:compMix -0.34 0.17 304 -2.07 0.04

genLV:day -0.03 0.02 304 -1.48 0.14

Correlation:

(Intr) genLV compMix day genLV:compMix

genLV -0.63

compMix -0.59 0.40

day -0.68 0.33 0.10

genLV:compMix 0.47 -0.80 -0.61 -0.07

genLV:day 0.39 -0.53 -0.01 -0.57 0.090

Standardized Within-Group Residuals:

Min Q1 Med Q3 Max

-1.70 -0.85 -0.064 0.75 2.65

Number of Observations: 368

Number of Groups: 60

**Table E: Statistical results shedding intensity analysis experiment 2.**

Linear mixed-effects model fit by REML

AIC BIC logLik

1408.37 1456.32 -693.19

Random effects:

Formula: ~1 | fish

(Intercept) Residual

StdDev: 1.95e-05 0.22

Correlation Structure: AR(1)

Formula: ~1 | fish

Phi parameter estimate: 0.47

Variance function:

Structure: Power of variance covariate

Formula: ~fitted(.)

Power parameter estimates: 0.89

Fixed effects: log.vl ~ gen + comp + day + gen:comp + gen:day + comp:day

Value Std.Error DF t-value p-value

(Intercept) 4.81 0.12 520 38.43 0.00

genMER95 0.18 0.18 520 1.00 0.32

compMix 0.52 0.19 58 2.73 0.01

day -0.05 0.01 520 -5.40 0.00

genMER95:compMix -0.47 0.18 520 -2.62 0.01

genMER95:day -0.03 0.01 520 -2.04 0.04

compMix:day -0.05 0.02 520 -2.58 0.01

Correlation:

(Intr) gen comp day gen:comp gen:day comp:day

genMER95 -0.60

compMix -0.60 0.30

day -0.72 0.37 0.40

genMER95:compMix 0.49 -0.81 -0.41 -0.21

genMER95:day 0.29 -0.52 -0.01 -0.41 0.12

compMix:day 0.28 -0.05 -0.75 -0.39 0.11 -0.06

Standardized Within-Group Residuals:

Min Q1 Med Q3 Max

-2.34 -0.81 -0.08 0.66 3.34

Number of Observations: 585

Number of Groups: 60

**Table F: Statistical results shedding intensity analysis experiment 3.**

Linear mixed-effects model fit by REML

AIC BIC logLik

821.40 840.23 -405.70

Random effects:

Formula: ~1 | fish

(Intercept) Residual

StdDev: 3.60e-05 0.91

Correlation Structure: AR(1)

Formula: ~1 | fish

Phi parameter estimate(s): 0.38

Fixed effects: log.vl ~ day

Value Std.Error DF t-value p-value

(Intercept) 5.00 0.10 280 50.30 0

day -0.07 0.01 280 -7.40 0

Correlation:

(Intr)

day -0.71

Standardized Within-Group Residuals:

Min Q1 Med Q3 Max

-1.91 -0.81 -0.14 0.70 3.03

Number of Observations: 321

Number of Groups: 40

**Table G. Statistical results total virus shed experiment 1 peak period.**

Linear mixed-effects model fit by REML

AIC BIC logLik

85.16 99.14 -36.58

Random effects:

Formula: ~1 | fish

(Intercept) Residual

StdDev: 0.40 0.16

Fixed effects: log10(tot) ~ gen + comp + gen:comp

Value Std.Error DF t-value p-value

(Intercept) 5.15 0.10 58 53.46 0.00

genLV 0.04 0.14 18 0.28 0.79

compMix 0.02 0.14 58 0.14 0.89

genLV:compMix -0.44 0.15 18 -3.05 0.01

Correlation:

(Intr) genLV compMx

genLV -0.71

compMix -0.71 0.50

genLV:compMix 0.66 -0.94 -0.53

Standardized Within-Group Residuals:

Min Q1 Med Q3 Max

-2.34 -0.30 0.03 0.25 2.69

Number of Observations: 80

Number of Groups: 60

**Table H. Statistical results total virus shed experiment 2 peak period.**

Linear mixed-effects model fit by REML

AIC BIC logLik

57.72 67.15 -24.86

Random effects:

Formula: ~1 | fish

(Intercept) Residual

StdDev: 0.27 0.21

Fixed effects: log10(tot) ~ comp

Value Std.Error DF t-value p-value

(Intercept) 6.27 0.05 58 116.68 0.00

compMix -0.29 0.09 58 -3.28 0.002

Correlation:

(Intr)

compMix -0.62

Standardized Within-Group Residuals:

Min Q1 Med Q3 Max

-1.46 -0.43 -0.05 0.36 1.94

Number of Observations: 80

Number of Groups: 60

**Table I. Statistical results total virus shed experiment 3 peak period.**

lm(formula = log10(tot) ~ gen)

Residuals:

Min 1Q Median 3Q Max

-1.04 -0.22 -0.02 0.25 1.27

Coefficients:

Estimate Std. Error t value Pr(>|t|)

(Intercept) 5.90 0.10 61.78 < 2e-16 ***

genLR80 -0.38 0.14 -2.79 0.008 **

---

Signif. codes: 0 ‘***’ 0.001 ‘**’ 0.01 ‘*’ 0.05 ‘.’ 0.1 ‘ ’ 1

Residual standard error: 0.43 on 38 degrees of freedom

Multiple R-squared: 0.17, Adjusted R-squared: 0.15

F-statistic: 7.77 on 1 and 38 DF, p-value: 0.0083

**Table J. Statistical results total virus shed experiment 1 post-peak period.**

Family: nbinom2 ( log )

Formula: tot ~ gen + (1 | fish)

Zero inflation: ~gen

AIC BIC logLik deviance df.resid

1232.9 1247.0 -610.5 1220.9 71

Random effects:

Groups Name Variance Std.Dev.

fish (Intercept) 0.85 0.92

Number of obs: 77, groups: fish, 58

Overdispersion parameter for nbinom2 family (): 1.47

Conditional model:

Estimate Std. Error z value Pr(>|z|)

(Intercept) 9.76 0.26 38.29 < 2e-16 ***

genLV -1.95 0.33 -6.00 1.93e-09 ***

---

Zero-inflation model:

Estimate Std. Error z value Pr(>|z|)

(Intercept) -2.17 0.53 -4.11 3.96e-05 ***

genLV 2.06 0.62 3.33 0.00087 ***

---

Signif. codes: 0 ‘***’ 0.001 ‘**’ 0.01 ‘*’ 0.05 ‘.’ 0.1 ‘ ’ 1

**Table K. Statistical results total virus shed experiment 2 post-peak period.**

Family: nbinom2 ( log )

Formula: tot ~ gen + (1 | fish)

Zero inflation: ~gen

AIC BIC logLik deviance df.resid

1773.2 1787.5 -880.6 1761.2 74

Random effects:

Groups Name Variance Std.Dev.

fish (Intercept) 0.77 0.88

Number of obs: 80, groups: fish, 60

Overdispersion parameter for nbinom2 family (): 1.45

Conditional model:

Estimate Std. Error z value Pr(>|z|)

(Intercept) 12.27 0.24 51.40 <2e-16 ***

genMER95 -3.07 0.26 -11.83 <2e-16 ***

---

Signif. codes: 0 ‘***’ 0.001 ‘**’ 0.01 ‘*’ 0.05 ‘.’ 0.1 ‘ ’ 1

Zero-inflation model:

Estimate Std. Error z value Pr(>|z|)

(Intercept) -20.07 3611.00 -0.01 0.996

genMER95 18.97 3611.00 0.01 0.996

**Table L. Statistical results total virus shed experiment 3 post-peak period.**

Family: nbinom1 ( log )

Formula: tot ~ gen

Zero inflation: ~gen

AIC BIC logLik deviance df.resid

640.4 648.9 -315.2 630.4 35

Overdispersion parameter for nbinom1 family (): 6.35e+04

Conditional model:

Estimate Std. Error z value Pr(>|z|)

(Intercept) 11.34 0.23 48.78 <2e-16 ***

genLR80 -0.60 0.33 -1.82 0.07.

---

Signif. codes: 0 ‘***’ 0.001 ‘**’ 0.01 ‘*’ 0.05 ‘.’ 0.1 ‘ ’ 1

Zero-inflation model:

Estimate Std. Error z value Pr(>|z|)

(Intercept) -0.41 0.46 -0.89 0.37

genLR80 -0.001 0.65 -0.001 0.999
