# Supplementary material for: Virus shedding kinetics and unconventional virulence tradeoffs
Source: PLoS Pathog. 2021 May 10;17(5):e1009528. doi: 10.1371/journal.ppat.1009528 (PMC8109835; doi:10.1371/journal.ppat.1009528)
Supplement: Attachment — Submitted filename: ResponseToReviewersFinal.pdf [file ppat.1009528.s002.pdf]

Dear editor,

We would like to thank you and the three anonymous reviewers for your insightful comments on our manuscript. We are excited to hear that you found the manuscript of interest and believe it would make an important contribution. We have addressed all of the reviewer comments as discussed below. We hope that you find the manuscript now suitable for publication. Please note that the page numbers given below apply to the version of the manuscript showing markups.

Major Comments

The reviewers only raised two major comments with our manuscript as follows.

1) Reviewer 2 inquired as to why each experiment was analyzed separately, rather than pooling the experiments into one large analysis.

Thank you for this suggestion. We have added text to lines 634-638 addressing this. In more direct response to the reviewer, they are correct that experimental effects are frequently observed from *in vivo* studies. This is true for IHNV in rainbow trout and has been demonstrated by our group as well as others. There are likely a myriad of host and environmental factors which could contribute to these experimental effects, despite the fact that environment was highly controlled in our experiments to minimize experiment effects. The most common phenomenon is that virulence and total shedding will vary for a given IHNV genotype between experiments. This was true here, for example genotype HV had higher virulence in experiment 1 compared to experiment 3. Despite these upward or downward shifts in absolute virulence or shedding, the relative differences between genotypes is quite reliably consistent between experiments. In other-words, virulence of all genotypes may shift upwards from one experiment to the next, but genotype 1 will have greater virulence than genotype 2 in both experiments. These relative differences were what we were most interested in quantifying here, rather than estimating absolute virulence or fitness, which will depend on a variety of environmental factors in any biological system. Ultimately, because of these experiment effects, it is not possible to pool all data from each experiment into one analysis. Such pooling would assume that the phenotype of the virus genotype (here virulence or shedding) was the same across experiments. A more practical reason for not combining all experiments into one analysis is that they were not fully factorial. For example, the viral genotypes, sampling days, and inclusion of mixed infections were not exactly the same in each experiment. As such, we have elected to analyze each experiment separately to avoid inaccurate statistics and reaching false conclusions. We believe that this is the most transparent and conservative approach for analyzing the data while acknowledging experiment effects. The exception to this is for our virulence fitness trait association section (Fig. 5), where we combined all experiments into one analysis in an effort to address the reviewer's comments (lines 675-689).

2) The reviewer also asked how mortality observed in these experiments would reflect natural mortality in the field.

Thank you again for an insightful comment. We have added text to lines 535-538 of the manuscript to address this. As discussed above, fish mortality and viral virulence can depend on

an array of environmental conditions. Again, here we were interested in quantifying relative differences between genotypes under controlled conditions, rather than absolute virulence or fitness values. We believe that these relative differences would be consistent in the field and across differing environments. Although it is impossible to know this with certainty, we would argue that this is true with any experimental study, or even field study, given the myriad of environmental factors that could be at play. We believe the results are of value in the context presented, but acknowledge the limitations of extrapolating laboratory data to field inference. As a reassurance, our group has conducted a large number of studies (many published and cited throughout the manuscript) quantifying the virulence and fitness of the IHNV genotypes investigated here, and we observed consistent patterns.

Minor Revisions

Reviewer #1:

1. Results: when reading the results I wondered why the data were not analyzed while excluding the zeroes (fish that did not die). This is later mentioned in the methods, but I think it would be worth mentioning this in the results already, and potentially showing best fit relationships for both sets of data (with and without fish that did not die).

We have added additional text to lines 189-195, 261-262, 267-268, 413-419, and 639-640 of the manuscript addressing this. Briefly, we did explore analysis of the data both with exclusion and inclusion of dead fish. This did not change the statistical outcome. Given the large number of complex statistical analyses conducted, we felt it would be distracting to the reader to present both results, since the same statistical conclusions were reached. As such, we have elected to only show data where dead fish were excluded (24 hours after death and onwards). However, we have more fully clarified this in the text in the places noted above.

2. Lines 298-299. When reading this section, I wondered why only an analysis of individual fish was presented. What would relationships across the experiments look like if data were analyzed on a per-genotype basis?

This question was also asked by reviewer 2 in the major comments section and addressed there.

3. Line 359: were trade-offs really observed? Or were positive relationships observed? Trade-offs are really negative relationships. In the trade-off hypothesis, the trade-off arises because virulence increases transmission rate while reducing the transmissible period.

We have changed the wording here to "Although virulence covaried with other traits, our observations did not conform to conventional theory" to address the reviewer's comment (now line 387). We agree that conventional virulence tradeoffs were not observed in this study and thank you for this recommended clarification. Classical tradeoff theory ascertains that high virulence comes with the virus fitness benefit of increased transmission rates but the cost of shorter transmission durations. We did not observe a consistent pattern of more virulent genotypes having higher transmission rates. Furthermore, we observed that more virulent genotypes had longer transmission durations and lower recovery rates (the opposite of classical theory). There was some evidence that high virulence had costs, in that host death did truncate transmission duration slightly, but it was drastically outweighed by recovery. As such, the

traditional transmission duration truncation cost of virulence was not apparent in our data. However, virulence was more likely to result in host culling, which is a cost of high virulence if it occurred, because it reduced transmission duration. As such, culling created a fitness tradeoff, but this was a novel insight outside of classical virulence tradeoff theory.

Reviewer #2:
The manuscript addresses a number of different questions using the same set of experiments, including mixed infections and their effects on virulence. I didn't see any mention of mixed infections (or culling effects) in the introduction, which made it feel a bit out of nowhere when the results were presented. Could some rationale / context for those aspects of the study be incorporated into the introduction? Relatedly, given the format that PLoS uses with the methods at the end, a few more details about the experiments should be presented either at the start of the results or the end of the intro (in particular, I was wondering whether the dose used in mixed infections was identical to those for single infections, so that seems like a key detail to have before the results are presented). I also had trouble figuring out the motivation for the mixed infection studies given that they are thought to be uncommon in the field. Perhaps this could be set up in the intro.

We have added text to lines 144-149 addressing this as suggested by the reviewer.

In the discussion, I wondered if the authors might be able to speculate about why some systems might conform to the classical trade-off model but not others. This may not be something they feel comfortable doing at this point, but I think the results are important and I wondered if there are certain characteristics of the system (water-borne pathogen, etc.) that may make it more likely to fall outside of the traditional trade-off model?

We have added text to lines 558-560 of the manuscript addressing this. The reviewer is correct that this is an important question and beyond the scope of the manuscript to discuss completely. However, we made an attempt at addressing it in a concise way, noting the very acute nature of the viral shedding in IHNV as a possible explanation in our specific case.

Line 51: a word "in" is missing here after "resulting".

Fixed.

Line 78: This may be personal preference but this paragraph is really long. Might help to start a paragraph break at "In recent years."

Fixed, now line 79.

Line 110: an "of" is missing after "fitness traits"

Fixed, now line 112.

Line 186: again this may be personal preference, but I found the phrasing awkward - essentially

a double negative. Could you say instead "with a more rapid decline in probability of shedding for the less virulent genotype..."

Fixed, now line 198-199.

Line 249: I assume you mean "on average" when you say "by all 20 fish"? I would clarify because when I first read it I thought that you had summed the data across all 20 fish.

We apologize for the confusion regarding this. We summed the total amount of virus shed for each individual fish over the course of the experiment to compare total virus shed between treatments. We have clarified this working on line 265 of the manuscript.

Line 255: minor but I don't think you need the words "the data analysis revealed" since it's implied

Changed, now line 275.

Lines 261-263: it took me awhile to figure out how this result differed from the one presented above. This section could benefit from adding extra text to clarify the distinction between the various questions asked. I wondered if perhaps even separating out the mixed infection questions from the virulence questions would help structure the results better. I struggled to follow all of the various results.

We agree the results presented in this section are complex, although they are accurate when referring to Figure 4A. We have clarified this section by some simplification on line 282. We considered separating the mixed infection from the single infection results, but this did not provide for easier comprehension because it is essential to compare the single versus mixed infections in order to assess competitive suppression. This made the results repetitive and did not seem to improve comprehension, so we did not re-structure the section.

Line 292: is "transmission rate" here akin to shedding? I think you define it later but I would be clear upon first use about what you mean.

The term has now been defined in this section as requested, now on lines 317-318.

Line 298: I would add "viral" before fitness both at the end of line 298 and in the next sentence in line 299, just to clarify that it is viral rather than fish fitness you are referring to. Similarly for line 311.

All fixed, now lines 323 and 337

Line 315. "parameters" should be "parameterized"

Fixed, now line 341.

Line 336: personal preference but I suspect adding a comma after "culling" for clarity

Changed as suggested, now line 364.

Lines 545-548: this all seem like key details for interpretation of the results- could they be moved to to the start of the results?

We have added text to lines 145-148 to address this.

Line 627: should "at" be "as"?

Fixed, now line 681.

Line 638: I think "from" is meant to say "farm"

Fixed, now line 694.

Figure 1. The panels are very small- this may just be the case for review but they are difficult to read at the current size. I wonder if you could use color coding that better corresponds to strain virulence somehow, to make interpretation of the graphs a bit easier.

We apologize for the small figures. This is likely a result of the online submission system. We will recommend to the editor that the figures be enlarged for the printed publication. We have carefully color coded our figures so that a given genotype has the same color in each figure to assist with clarity.

Figure 5. Given that these lines are not statistically significant, I would make them dashed instead of solid.

Thank you for this suggestion. We are concerned a dashed line would be difficult to see by the reader and have elected to keep this as a solid line. However, we have clarified in the figure legend that the lines are not significant (line 335-336).

Reviewer #3:
P14L212 and P11L224: Suggest also to refer to fig 3 in addition to Tables.

Fixed, now line 227.

P15L326 and 328: Please check if correct fig references are used

Fixed, now lines 354 and 356.

P18L401: Please check if correct fig references are used (e.g. there are no Fig 4D in the manuscript)

Fixed, now line 438.

P9 - "Number of fish shedding"-section and P54 - Fig 2
Materials and methods chapter (P26L561) described that dead fish were left in their tanks and that water samples were continued to be taken for the full duration of the experiment. While reading the "Number of fish shedding"- section, I was starting to wonder how these results was (or wasn't) included in e.g. measurements of viral shedding. I eventually found in figure legends that these results were only included using the sampling at 24 hours after death. I suggest some information in the text part as well on which samplings (i.e. live fish only) the results are based on.

We have added text to lines 189-195, 215, 225, 261-262, 265, and 413-419 addressing this.

Regarding Fig. 2 and e.g. statements in line 180-182 ("Shedding then gradually decreased and by day 15 the majority of fish had stopped shedding. However, a small number of fish (1-5), were still shedding detectable virus at the end of each experiment (day 30-35)."): I started to question myself if "the majority of fish" weren't dead by day 15 (at least for some isolates)? I suggest it could be clearer if the "Number of fish" in Fig 2 and "majority of fish" in text were replaced and presented to relate to the remaining live fish only (e.g. "Percent of live fish" or similar). The reader will get an impression on how many live fish there are left at each time points from fig 1, but it is hard to relate that precisely to fig 2 and results description. "Percent live fish" is used in an example mentioned in Discussion chapter (P17L366-369) and I would like similar presentation to be used in Fig. 2 and related text as well.

We have changed the text in line 189 to "remaining live fish" as suggested. We elected to keep figure 2 as number of fish rather than percent of fish. This is because the number in this figure also provides the number of fish used for statistical analysis and mean values presented in figure 3. However, we have clarified the figure legend to more explicitly reflect this (line 215).

P10 – "Shedding intensity"-section
Similar as described above, is also related to this section where line 210-211 states that "Afterwards shedding intensity remained stable, although the number of fish shedding continued to decrease". Please relate/correct this to relate to the number of live fish.

We have changed this as suggested, now line 225.

In Fig 3 legends it is stated (L245-246) that "Number of fish included in the mean values can be found in figure 2.", but I cannot easily find such information in Fig 2 (but would definitely like to have it).

In addition to the changes made to figure 2 legend discussed above, we added text to figure 3 legend (lines 261-262) to further clarify this.

P12 – "Total virus shed"-section
L249 "..the total amount of virus shed by all 20 fish in each treatment group over the course of infection" - Please specify how this relates to live fish as it can be interpreted that you include the amount "shed" (i.e. released) from decomposed fish tissue of dead fish (available data on this is shown in fig S1.)

Clarified as suggested with additional text added to lines 267-268.

MATERIALS AND METHODS chapter
A lot of the methodology is well described previously, and the authors refer to previous publications describing it. This is good and increase quality as the methods have been used earlier, but for the reader it would be easier if also a SHORT description of the basics was included, where applicable. Please consider.

We have added some details to the methods where appropriate. These additions can be found on lines 610-619.

SUPPLEMENTARY
Mat & Met chapter P26 and Fig S1: Mat & Met describes that "dead fish were left in their tanks and continued to be sampled for the duration of the experiments (30-35 days)". The result of these analyses of virus presence in the water is shown in fig S1, but is not commented or discussed anywhere. I would like to know the intention of including this and also that the results were commented in the manuscript. It seems that (by a rough overview) e.g. that fish infected with high virulent isolates release virus for quite a long time post mortem (probably to a large extent due to decomposing of tissue as it can not be defined as true "shedding" due to replicating virus). These results are probably not relevant for the discussion of trade-off theory in the manuscript, but if included it is worth a comment.

This has been addressed in response to previous reviewers' comments. In brief, we have added lines 413-419 to the discussion section addressing the potential impact of shedding from dead fish on virulence evolution.

We again thank the reviewers for their helpful suggestions. Please let us know if we can provide additional information.

Sincerely,

Andrew Wargo

Andrew Wargo
(on behalf of all authors)
